# Facial expressions and eye tracking in individuals with social anxiety disorder: a systematic review

**DOI:** 10.1186/s41155-019-0121-8

**Published:** 2019-04-11

**Authors:** Rianne Gomes e Claudino, Laysa Karen Soares de Lima, Erickson Duarte Bonifácio de Assis, Nelson Torro

**Affiliations:** Graduate Program in Cognitive and Behavioral Neuroscience, Federal University of Paraíba – UFPB, João Pessoa, 58051-900 Brazil

**Keywords:** Social phobia, Facial expressions, Eye tracking, Eye movements

## Abstract

Social anxiety disorder (SAD) is characterized by the fear of being judged negatively in social situations. Eye-tracking techniques have been prominent among the methods used in recent decades to investigate emotional processing in SAD. This study offers a systematic review of studies on eye-tracking patterns in individuals with SAD and controls in facial emotion recognition tasks. Thirteen articles were selected from the consulted databases. It was observed that the subjects with SAD exhibited hypervigilance-avoidance in response to emotions, primarily in the case of negative expressions. There was avoidance of conspicuous areas of the face, particularly the eyes, during observations of negative expressions. However, this hypervigilance did not occur if the stimulus was presented in virtual reality. An important limitation of these studies is that they use only static expressions, which can reduce the ecological validity of the results.

## Background

A systematic review was undertaken of studies that use eye-tracking techniques to observe emotional facial expressions in individuals with SAD and their respective controls. We sought to identify whether there are distinct visual scanning and eye fixation patterns that are characteristic of SAD.

## Main text

### Introduction

The DSM-5 includes social anxiety disorder (SAD) or social phobia in the list of anxiety disorders as a specific phobia type. The primary symptom is the patient’s strong fear of being judged negatively, particularly in social situations that involve interaction with strangers or being exposed to others during an activity such as speaking, eating, or drinking in public. A person with SAD may feel embarrassed, humiliated, rejected, or fearful of offending someone as a result of his or her behavior (American Psychiatric Association, 2013).

According to Furmark (2002), the prevalence of this disorder in Western countries ranges from 7 to 13% throughout the lifespan, making it one of the most prevalent psychiatric disorders worldwide. In Brazil, it is estimated that 11.6% of young college students are affected by SAD but rarely seek help, as they believe that the symptoms are intrinsic to their personality (Baptista et al., 2012). SAD is considered a chronic, disabling disorder that impairs social interaction and causes losses in academic, professional, personal, and emotional areas (Arrais et al., 2010; Kaplan, Sadock, & Grebb, 2003).

Because the correct interpretation of facial expressions is important in social communication and interpersonal relationships, facial expressions are used as experimental stimuli to understand the biases in social information processing in social anxiety (Coles, Heimberg, & Schofield, 2008). According to Matsumoto, Keltner, Shiota, O’Sullivan, & Frank (2008), facial expressions are related to emotional experience and fundamental to an individual’s adaptation to his or her social environment because they provide clues regarding how to interact with others.

Most studies have reported that individuals with SAD have greater acuity in the recognition of negative emotions (Coles & Heimberg, 2005; Foa, Gilboa-Schechtman, Amir, & Freshman, 2000; Machado-de-Sousa et al., 2010). Other studies indicate that there is no misinterpretation of emotional facial expressions in SAD or any clear bias with respect to emotional expressions (de Jong & Martens, 2007; Philippot & Douilliez, 2005).

The evaluation of eye movements, also known as *eye tracking*, is a prominent method used to study emotional processing and the observer’s visual and cognitive processes (Salvucci & Goldberg, 2000; Vandeberg, Bouwmeester, Bocanegra, & Zwaan, 2013). This approach became more widespread with the emergence of the “*strong eye-mind*” hypothesis, formulated by Just and Carpenter (1980), according to which there is no delay between what is fixated on and the cognitive process associated to the fixation.

Eye-tracking studies demonstrate the presence of attentional biases and different eye-movement patterns in persons with SAD when observing a face. Generally, there is first a hypervigilance regarding negative stimuli, followed by an avoidance of such stimuli (Armstrong & Olatunji, 2012; Liang, Tsai, & Hsu, 2017; Weiser, Pauli, Weyers, Alpers, & Mühlberger, 2009b). In healthy individuals, eye movements demonstrate a pattern of fixation on facial regions in an inverted triangle shape that includes the eyes, nose, and mouth because a greater amount of facial information is found in those areas (Mertens, Siegmund, & Grüsser, 1993). However, whether these patterns occur regardless of the displayed emotion and stimulus presentation time or whether they vary according to stimulus type has not been established.

The objective of the present study was to perform a systematic review of studies that evaluated the patterns of ocular movement during the evaluation of emotional facial stimuli in individuals with social anxiety disorder. We chose not to conduct a meta-analysis because of the methodological diversity of the eye-tracking research, which vary in relation to the type of emotion presented, exposure time of the stimuli, samples of the study, algorithms for recording and analyzing data, among other aspects, which could hamper the integration of results. In addition, there are other problems inherent to the meta-analysis procedure, such as the occurrence of non-linear correlations, multifactorial effects (instead of unifactorial), and non-homogeneous data disconnected from the hypothesis (Greco, Zangrillo, Biondi-Zoccai, & Landoni, 2013).

This review presents guidelines based on the Preferred Reporting Items for Systematic Reviews and Meta-Analyses (PRISMA) initiative (http://www.prisma-statement.org/) for bibliographic research and data communication in systematic reviews. The review was registered in the *international prospective register of systematic reviews* (PROSPERO) database under the title “Facial Expressions and Eye Tracking in Social Anxiety Disorder: A Systematic Review” and is in the analysis phase awaiting registration confirmation.

### Literature search

A systematic literature search was performed on the electronic databases PubMed, *ScienceDirect*, EBSCO, *Scopus*, *Web of Science*, and MEDLINE, considering publications since 1988, when LC Technologies, Inc., introduced the world’s first computer-based eye-tracking system (Barreto, 2012). The end date for the search was 4 December 2017. A gray literature search was performed by analyzing studies present in the reference sections of the articles that comprise this review’s sample. However, digital libraries of theses and dissertations were disregarded. The descriptors shown in Table 1 were used for the study search, and the search strategies for each database can be found in that table.

**Table 1:** Search strategies by database

PubMed	(“social anxiety” OR “social phobia”) [Title/Abstract] AND (“eye tracker” OR “eye tracking” OR “eye movements” OR “eye gaze” [Title/Abstract]) AND (“emotional face” OR “emotion” OR “face” OR “facial expression” OR “facial emotion” OR “facial” OR “emotional facial expression” [Title/Abstract])
Web of Science	(TI = (social anxiety OR social phobia) AND TI = (eye tracker OR eye tracking OR eye movements OR eye gaze) AND TI = (emotional face OR emotion OR face OR facial expression OR facial emotion OR facial OR emotional facial expression))
Scopus	TITLE-ABS-KEY(“anxiety” OR “social phobia”) AND TITLE-ABS-KEY(“eye tracker” OR “eye tracking” OR “eye movements” OR “eye gaze”) AND TITLE-ABS-KEY(“emotional face” OR “emotion” OR “face” OR “facial expression” OR “facial emotion” OR “facial” OR “emotional facial expression”)
EBSCO	(AB “social anxiety OR social phobia”) AND (AB “eye tracker OR eye tracking OR eye movements OR eye gaze”) AND (AB “emotional face OR emotion OR face OR facial expression OR facial emotion OR facial OR emotional facial expression”)
ScienceDirect	(“social anxiety” OR “social phobia”) AND (“eye tracker” OR “eye tracking” OR “eye movements” OR “eye gaze”) AND (“emotional face” OR “emotion” OR “face” OR “facial expression” OR “facial emotion” OR “facial” OR “emotional facial expression”)
MEDLINE	Tw:(social anxiety OR social phobia AND eye tracker OR eye tracking OR eye movements OR eye gaze AND emotional face OR emotion OR face OR facial expression OR facial emotion OR facial OR emotional facial expression)

### Eligibility criteria

The article inclusion criteria were as follows: (1) full-text articles, so that all stages of the research can be accessed; (2) inclusion of an experimental task using facial expressions; (3) use of eye-tracking techniques; (4) inclusion of a sample that consisted of young adult subjects with social anxiety and no other disorder; (5) results that compared a control group (healthy) with a clinical one (with social anxiety); and (6) published in any language. Literature reviews, meta-analytical studies, theses, and dissertations were not included.

### Study selection

To select the studies, a database search was initially performed using the strategies included in Table 1. Two reviewers (EDBA, LKSL) participated in the initial search, independently evaluating the titles and abstracts of each article and selecting those with the potential to be included in this review. Then, RGC compared the results and deleted articles repeated between databases. The titles and abstracts were read, and manuscripts that were explicitly discordant in terms of the criteria and purpose of this study were also excluded (e.g., studies that used a sample of children or studies that investigated disorders other than social anxiety).

After selection, the full text of the selected manuscripts was read, and those that met the study’s eligibility criteria were identified. Because there was no disagreement regarding article selection, a consensus meeting was not required at this stage. Thirteen articles that used eye-tracking tasks with facial expressions to evaluate a young adult sample with social anxiety were included in the review.

### Data collection

After identifying the citations that comprised the review sample, the reviewers named above independentlyextracted the data to be analyzed in each article. The following variables were collected for each selectedarticle: authors and year of publication, number of participants, equipment used, type of stimuli used,emotions, applied method, presentation time, and main results. This information is shown in Table 2.

**Table 2:** Studies selected for the review according to author, sample description, type of stimuli, and emotions

Authors	Sample	Apparatus	Type of stimuli	Emotions	Method	Time	Main results
Horley et al. (2003)	Clinical SAD patients (*n* = 15); controls (*n* = 15)	CEDRIC Mark II	Photographs	Neutral, happy, and sadness	Required to look at the fixation point for 1 s, just until the face appeared. Afterward, could freely look at the face	10 s	Subjects with SAD: Lack of fixation for sadness and neutral; greater tracking length; tendency to avoid fixation on eyes, nose, and mouth; avoidance of eyes on sad faces
Horley et al. (2004)	Clinical SAD patients (*n* = 22); controls (*n* = 22)	CEDRIC Mark II	Photographs	Happy, sadness, anger, and neutral	Looked freely at faces	10 s	Subjects with SAD: Hypervigilance and avoidance of eyes in the case of anger
Garner et al. (2006)	Exp. 1: (*n* = 40) students (control and SAD); Exp. 2: (*n* = 40) students (control and SAD)	Eye Tracker and Gaze Tracker, Applied Science Laboratories, Model 504	Photographs of faces and domestic objects (e.g., chair, lamp, clock)	Neutral, happy, and anger	Exp. 1: Pair of images (neutral-emotion or neutral-object), subsequently replaced by two points (vertical or horizontal) in the position of one of the faces. Required to indicate the orientation of the points Exp. 2: Before Task 1 were instructed to present a speech to a camera	1.5 s	Exp. 1: Subjects with SAD: First fixation and maintenance in the case of emotional faces; longer fixation time on neutral faces than on objects Exp. 2: Subjects with SAD: Shorter fixation latency for emotional faces but with shorter fixation time Both groups: Fixation first and gaze maintained for longer for faces as opposed to objects
Mühlberger et al. (2008)	SAD students (*n* = 12); controls (*n* = 14)	iView X Hi-Speed, SMI	Virtual environments with elevator, person, or virtual object (e.g., bookcase)	Anger and happy	In an elevator with opening doors (on 60 floors) pairs of stimuli (two people with different expressions, one happy person and a bookcase, one angry person and a bookcase) were presented	6 s	Subjects with SAD: Initially avoided the faces and avoided maintaining fixation on angry faces
Weiser et al. (2009b)	*n* = 29 female students (separated into 2 groups)	iView X Hi-Speed, SMI	Virtual photographs	Happy, anger, and neutral	Explored a pair of faces (neutral-emotion). Afterward, judged the valence and arousal of the face	3 s	Subjects with SAD: Hypervigilance in the first fixation in the case of emotions; attentional bias toward happy female faces; modest hypervigilance-avoidance regarding emotions
Weiser et al. (2009a)	Students with high levels of SAD (*n* = 21); low levels of SAD (*n* = 21); controls (*n* = 20)	iView X Hi-Speed, SMI	Virtual photographs	Happy, anger, sadness, fear, and neutral	Faces presented at the sides of the screen. Required to perform prosaccades or antisaccades toward the faces, then judge the valence and arousal of the face	1 s	Subjects with SAD: Antisaccades with more errors in response to all facial expressions Both groups: Correct antisaccades with more time in response to fearful faces
Moukheiber et al. (2010)	SAD patients (*n* = 26); control (*n* = 24)	EyeLink II	Male and female photographs	Happy, surprise, disgust, sadness, anger, fear, and neutral	There was no participant task; subjects were required to hold the head still and, after cross-calibration in the middle of the screen between the pictures, to look at the pictures	10 s	Subjects with SAD: Hyperscanning overview and a reduction in fixations and time for the eye region and to specific emotions, most notably anger and disgust. No difference was observed in relation to gaze avoidance according to the correspondence of the sex of the subject and that of the image
Lange et al. (2011)	SAD students (*n* = 22); controls (*n* = 21)	EyeLink V02.01	Photographs	Anger, neutral, and happy	Explore matrices of neutral-angry or happy-angry faces. Had to judge the matrices as friendly or not	500 ms or 2.5 s	Subjects with SAD: Fixation on angry faces. Long presentation time: Quicker deviation if the original fixation was toward anger
Schofield et al. (2013)	Clinical SAD patients (*n* = 19); controls (*n* = 20)	EyeLink 1000 - SR Research	Photographs	Happy, fear, anger, and neutral	Pairs of facial expressions (anger-neutral, fear-neutral, happy-neutral) replaced by a down or up arrow in the position of one of the faces. Required to indicate the arrow type	1.5 s	Subjects with SAD: Similar fixation pattern toward emotion and neutral Controls: More fixation toward happy in the last moments of the presentation and less throughout the presentation in relation to negative emotions Two groups: Lower fixation latency for emotional faces
Finch et al. (2016)	SAD students (*n* = 36); controls (*n* = 37)	Tobii T120 eye-tracking system	Photographs	Anger, neutral, and happy	Looked at pairs of facial expressions for 3000 ms in two stages: during the first 500 ms of exposure and during the remaining time	3 s	Subjects with SAD: Initial bias toward social threat. In particular, socially anxious participants in the fear of death condition were vigilant in the detection of angry and happy faces
Boll et al. (2016)	Clinical SAD patients (*n* = 22); controls (*n* = 22)	EyeLink 1000	Grayscale photographs	Anger, fear, happy, and neutral	Exp 1: Rated the emotion of facial stimuli as quickly and accurately as possible Exp 2: Identified the target letter presented next to the facial stimuli as quickly and accurately as possible	150 ms or 3 s	Exp 1: Patients with SAD: Hypervigilance in relation to the mouth area regardless of the type of emotional expression. There was no evidence of subsequent avoidance of looking toward the eye. Time difference in looking toward the eye between patients and controls. Exp 2: Patients with SAD were significantly slower than controls in identifying the target letter, but there was no significant difference with respect to the number of correct responses when identifying letters
Kim and Lee (2016)	SAD students (*n* = 22); controls (*n* = 22)	iView X RED-IV, SMI	Face-body composites: consistent (same emotion) and inconsistent (different emotion)	Anger, fear, disgust, sadness, and happy	Looked at the picture and selected the emotional state that best described the presented individual	4 s	Individuals with SAD: Hypervigilance without avoidance toward the face for consistent composite face and body images. There was an avoidance of faces without hypervigilance
Lazarov et al. (2016)	Students with low levels of SAD (*n* = 20); students with high levels of SAD (*n* = 20); clinical SAD patients (*n* = 20)	SMI BeGaze native software	Color photographs of 16 male and 16 female actors	Unpleasant and neutral expressions	Looked freely at each matrix in any way desired until it disappeared. Student groups repeated the session after 1 week	6 s	Session 1: All groups spent less fixation time on threatening faces than neutral faces. High SAD and clinical patient group: The fixation time was greater on threat than the low SAD group Session 2: High SAD group exhibited more fixation time on threat than the low SAD group. No significant difference was found in fixation time on neutral faces

To evaluate the publication quality, the following factors were analyzed: the precise description of the study design, eligibility criteria, sample characterization, sample size, description of the intervention, and applied stimuli.

### Results

**Fig. 1:**
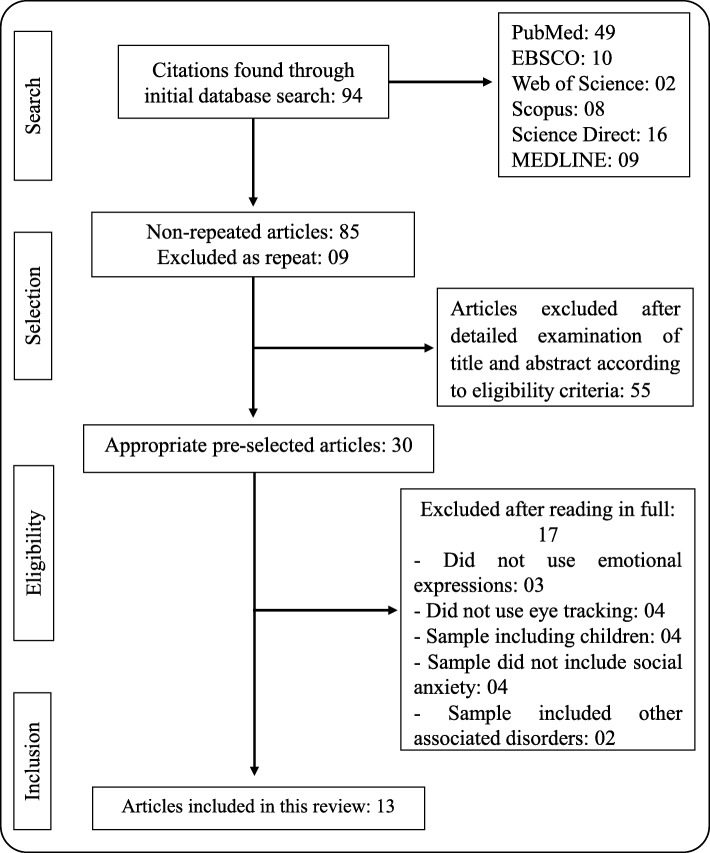
Stages of the search and selection process

The initial search performed on the databases with the strategies described in Table 1 resulted in the identification of 94 citations. After the removal of nine repeated articles, 85 publications remained. These publications were evaluated based on their titles and abstracts using the described inclusion criteria. Fifty-five articles were excluded at this stage, leaving 30. These studies were then read in their entirety, and 13 were selected as a result. The remaining 17 were excluded because they did not meet the inclusion criteria. No studies relevant to this review were found among the references analyzed in the gray literature. These data can be better comprehended by examining Fig. 1, which shows the literature search in detail.

Publications from 2003 to 2016 were found. There was not a substantial amount of variability in sample size among the selected studies. Two of the analyzed articles separated the samples into three groups (control/patients, low social anxiety, and high anxiety) (Lazarov, Abend, & Bar-Haim, 2016; Weiser, Pauli, & Mühlberger, 2009a). The remainder divided the samples into control and social anxiety groups. Six studies recruited clinical patients (Boll, Bartholomaeus, Peter, Lupke, & Gamer, 2016; Horley, Williams, Gonsalvez, & Gordon, 2003, 2004; Lazarov et al., 2016; Moukheiber et al., 2010; Schofield, Inhoff, & Coles, 2013), while others evaluated social anxiety in college students. Only one study exclusively focused on women (Weiser et al., 2009b).

The most commonly used eye-tracking devices were iView X (Kim & Lee, 2016; Mühlberger, Weiser, & Pauli, 2008; Weiser et al., 2009a; Weiser et al., 2009b) and EyeLink (Boll et al., 2016; Lange et al., 2011; Moukheiber et al., 2010; Schofield et al., 2013), both of which were used in four studies. Regarding the type of stimuli, all studies used static facial expressions. However, three studies employed virtually created faces (Mühlberger et al., 2008; Weiser et al., 2009a; Weiser et al., 2009b). All articles involved the presentation of at least one positive and at least one negative valence emotion except Lazarov et al. (2016), who did not use positive valence emotions. In addition, two studies did not include a neutral face (Kim & Lee, 2016; Mühlberger et al., 2008). Three publications used other stimuli, such as household objects and body images in addition to faces (Garner, Mogg, & Bradley, 2006; Kim & Lee, 2016; Mühlberger et al., 2008). Despite having different complementary tasks, the articles explained that these tasks served to keep the subjects’ attention.

The results obtained in the different studies were analyzed separately and are shown in Tables 2 in addition to the specific characteristics of each study.

### Discussion

SAD is characterized as a fear of social situations, in which the individual fears being judged negatively by others (American Psychiatric Association, 2013). Studies on facial expressions using eye-tracking techniques have gained importance in recent years. In this regard, this systematic review sought to identify whether there is a distinct pattern in the viewing of emotional facial expressions in individuals with SAD.

Horley et al. (2003) evaluated patients with SAD and controls in the free observation of emotional faces with negative, positive, and neutral valences for 10 s and found that individuals with social phobia had a different scanning strategy than the control group. Fixations were reduced in terms of amount and duration, and there was increased tracking of negative (sadness) and neutral expressions by subjects with SAD, along with an avoidance of fixations on conspicuous facial features (i.e., the eyes, the nose, the mouth) in the case of sadness. Thus, a negative stimulus provoked an avoidance response. A 2004 study by the same authors included an angry expression (referred to as threatening), and subjects with SAD were found to show hypervigilance, particularly toward the angry expression, and avoided the eye region in the case of this emotion. Therefore, the threatening face stood out compared to other stimuli.

Schofield et al. (2013) evaluated clinical patients with SAD and volunteer controls, presenting emotions in pairs. It was found that patients with social phobia exhibited a similar pattern of fixation on emotional and neutral expressions, while the control group had a tendency to avoid fixation on negative expressions (fear and anger) throughout the presentation and on positive (happy) ones toward the end of the presentation. In both groups, fixation latency was less for emotional faces than for neutral faces. Thus, it can be stated that the SAD subjects exhibited hypervigilance regardless of the emotional valence.

Similarly, Weiser et al. (2009b) analyzed women with social phobia during the free exploration of a pair of facial stimuli with different emotions. The participants with SAD displayed a hypervigilant pattern toward emotional faces irrespective of emotion, with an attentional bias toward happy female faces. A modest hypervigilance-avoidance pattern was found, whereby in the consecutive time interval, the women directed their attention to the neutral face.

Garner et al. (2006) combined photographs of faces (neutral, happy, and angry) with household objects, which could appear in a neutral-emotional or neutral-object pair. This study involved two experiments. In the first, the pair of images was presented for 1.5 ms, followed by two vertical or horizontal points displayed on the right or left. The subjects had to indicate the orientation type of the points. The second experiment included the addition of a social stressor. The subjects were instructed that they would have to present a speech to a camera on a subject defined moments before performing Task 1. In the first task, it was found that the subjects with SAD produced more fixations (amount and duration) toward the emotional and neutral faces than toward the objects. In the second task, subjects with SAD exhibited lower fixation latency in connection with the emotional faces compared to the neutral faces, but with a shorter fixation time. Thus, when a social stressor was added, only subjects with the disorder displayed increased vigilance toward the faces.

Moukheiber et al. (2010) evaluated the aversion of individuals with SAD compared to that of control subjects when looking at different emotional faces of men and women. The main findings were a significantly lower number of fixations and gaze length in patients with SAD as well as for each of the six basic emotions regardless of sex. In addition, a significant correlation was found between the severity of the phobia and the degree of visual avoidance. There was no difference in the pattern of avoidance according to the sex of the individual in the image. According to these authors, the results confirm and expand on previous results and suggest that visual avoidance is a central component of SAD pathophysiology and could be used as a behavioral phenotype in brain-imaging studies.

Investigating the effect of anger on stimuli that consisted of several faces (matrices or crowds), Lange et al. (2011) created 4 × 4 matrices with combinations of neutral and angry or angry and happy faces, presented for either a short (500 ms) or long (2.5 s) period of time. They found that the subjects with social anxiety had a tendency toward hypervigilance in response to the expression of anger. When the presentation time expired, the subjects with SAD deviated more quickly if the first fixation was toward an angry face. Thus, we can note a hypervigilance-withdrawal effect regarding that emotion.

Weiser et al. (2009a) evaluated participant saccade movements. Subjects had to produce prosaccades (looking toward) or antisaccades (looking away from) for faces presented for 1000 ms to the left or right of the screen. The researchers found that regardless of social anxiety, participants presented more prolonged latencies in the case of antisaccades (i.e., it took longer to produce this movement) and shorter latencies in the case of prosaccades in response to expressions of fear. This outcome suggests that the expression of fear seems to attract attention and is difficult to avoid voluntarily.

Mühlberger et al. (2008) created a virtual environment that featured a task closer to reality, with an ascending elevator that stopped at 60 floors. On each floor, the doors opened and one of three pairs of stimuli could be seen: (1) two people with different expressions (happy and anger), (2) a person with a happy expression and an object (a bookcase), or (3) a person with an angry expression and an object (a bookcase). The authors found that subjects with SAD initially avoided the facial regions and maintained their fixations on angry faces. These findings are contrary to previous studies that indicated hypervigilance regarding emotions, thus calling into question the ecological validity of those other studies.

Boll et al. (2016) demonstrated that patients with social phobia showed a clear hypervigilance toward the eye in relation to the mouth area. Participants were exposed to male and female faces on a grayscale (anger, fear, happy, and neutral). When a facial stimulus was presented, participants had to classify the emotion portrayed as quickly and accurately as possible by pressing one of four keys on a standard computer keyboard with the index or middle finger of both hands. Irrespective of the type of emotional expression, patients directed their attention first to the eyes more than controls, suggesting that automatic attentional orientation is more biased toward this region than others in individuals with social phobia. In addition, subjects with SAD looked longer at that region than controls.

Kim and Lee (2016) evaluated vigilance patterns using face-body images with consistent (same) and inconsistent (different) emotions. After a random display of 4000 ms, participants had to select the emotional state that best described the individual (happiness, sadness, anger, fear). It was found that individuals with social anxiety exhibited a complex pattern, displaying hypervigilance without avoidance toward the face in the case of consistent face and body images.

Finch, Iverach, Menzies, and Jones (2016) demonstrated that the fear of death could aggravate the anxious response in socially anxious individuals, with significant effects found for the initial bias toward a social threat. After completing a questionnaire that evaluated fear of death, 32 pairs of faces were presented to the participants with (1) angry and neutral and (2) happy and neutral facial expressions. Socially anxious participants in the experimental condition exhibited significantly more initial bias toward social threats than both non-socially anxious participants in the same condition and socially anxious participants in the control condition. In particular, socially anxious participants in the fear of death condition were vigilant in the detection of angry and happy faces.

In the search for reliable targets for therapeutic interventions, Lazarov et al. (2016) found that SAD is associated with an increased length of gaze at socially threatening stimuli when healthy students were compared with highly anxious students regarding eye-tracking patterns for neutral and threatening faces.

The studies present different methodological structures. Concerning the sample, five studies used a clinical sample (Boll et al., 2016; Horley et al., 2003, 2004; Moukheiber et al., 2010; Schofield et al., 2013) and eight recruited sample of students with symptomatology of SAD (Garner et al., 2006; Mühlberger et al., 2008; Weiser et al., 2009b; Weiser et al., 2009a; and Lazarov et al., 2016). Despite of this, no difference was found between the results of studies conducted with clinical samples compared to those conducted with students.

There was also diversity with regard to the type of emotion used as positive and negative stimulus. All studies presented happiness as a positive emotion; however, most of them used anger as a negative emotion, although others have also used sadness, fear, and disgust. Others analyzed the tracking of the neutral face. The results for positive, negative, and neutral valence showed a greater avoidance of negative emotions than positive ones. Furthermore, not all studies analyzed the tracking of facial regions (p.e., eyes, nose, and mouth).

A difference was found in the definition of fixation criteria, some attributed 100 ms, other 200 ms; few worked with speed, varying from 75°/s to 8000°/s^2^; and in four studies, the criteria established for the fixation was not clear (Finch et al., 2016; Kim & Lee, 2016; Mühlberger et al., 2008; Schofield et al., 2013). This could generate differences in the counting of the number of fixations in an image or region of interest and hinders the replication of the results (Salvucci & Goldberg, 2000).

Overall, the consulted studies found a pattern of hypervigilance-avoidance toward emotions for individuals with SAD. However, a limitation of the studies was the exclusive use of static stimuli (i.e., pictures). In everyday social relations, interaction conditions are significantly more complex, with dynamic facial expressions and changing emotional intensities. Studies that compare static and dynamic faces have shown that dynamic faces elicit greater activity in areas associated with the interpretation of social signals and the processing of emotions (Arsalidou, Morris, & Taylor, 2011; Recio, Sommer, & Schacht, 2011). Roy, Blais, Fiset, and Gosselin (2010) noted that healthy volunteers have different eye scanning patterns in response to static and dynamic faces.

Therefore, the use of dynamic expressions would enhance the ecological validity of these studies and bring them closer to the real situations of everyday social interaction (Alves, 2013; Torro-Alves, Bezerra, Claudino, & Pereira, 2013). Additionally, Torro-Alves et al. (2016) found that subjects with social phobia have an advantage in recognizing emotions when presented with less ecological validity (static faces) because the movement of facial expressions can mitigate or omit differences in the recognition of facial emotions between individuals with high and low social anxiety.

Another important methodological limitation found in the studies was the small sample size used in most of them, which may have had an influence on effect size. However, this influence would have to be verified by means of a meta-analysis.

### Limitations

This review has several potential limitations. First, there is the heterogeneity of the study participants (students and medical patients), which may have affected the ability to generalize the results, thus affecting the review’s external validity. Another limitation concerns the pre-defined methodological strategic restriction, which may have resulted in a failure to include relevant studies, such as theses and dissertations, excluded by the eligibility criteria. In addition, the risk of bias for randomized trials in the articles included in the study was not analyzed quantitatively or qualitatively. A meta-analysis of the studies found could offer relevant additional information on eye-tracking patterns in individuals with SAD.

### Conclusions

The studies analyzed in this review reveal that subjects with SAD have hypervigilance-avoidance effects in relation to emotions, primarily in relation to negative expressions (e.g., anger). This effect translates into a greater number of fixations in the first moments, followed by avoidance of gaze, particularly in the case of negative emotions, on the part of individuals with SAD. A preference for emotional faces over objects or neutral expressions was also observed. A study on the extent of eye movement revealed the avoidance of areas with conspicuous facial features, particularly the eye region, in the case of negative expressions. It was also noted that the introduction of a social stressor increased vigilance. However, when the stimulus was more realistic (e.g., virtual reality), this hypervigilance regarding faces was not found.

Although SAD is a highly prevalent disorder in society (Furmark, 2002), this field of study is a recently established one, the amount of information on eye tracking in the observation of facial expressions in individuals with SAD remains limited. These analyzed studies have methodological limitations, such as the exclusive use of static faces, which can reduce ecological validity because the study conditions do not closely match the real conditions of everyday social interaction. This limitation also affects the consistency of the findings. Nonetheless, no other systematic review on these themes had been done to the best of our knowledge.

It is recommended to carry out eye tracking studies using stimuli with greater ecological validity (dynamic faces, for example), which would allow to verify whether the pattern of hypervigilance remains the same in that condition. In addition, the fixation criteria chosen for data analysis must be better detailed. Studies with larger samples would increase the power of generalization of results. It is also recommended to carry out meta-analysis with more homogeneous subsets of studies that evaluate the recognition of facial emotions in social anxiety.

## Abbreviations

DSM-5: Diagnostic and Statistical Manual of Mental Disorders-5
SAD: Social anxiety disorder
